# Study protocol for the Heads-Up trial: a phase II randomized controlled trial investigating head-up tilt sleeping to alleviate orthostatic intolerance in Parkinson’s Disease and parkinsonism

**DOI:** 10.1186/s12883-023-03506-x

**Published:** 2024-01-02

**Authors:** Amber H. van der Stam, Nienke M. de Vries, Sharon Shmuely, Daan Smeenk, Joost H. Rutten, Ineke A. van Rossum, Susanne T. de Bot, Jurgen A. Claassen, Bastiaan R. Bloem, Roland D. Thijs

**Affiliations:** 1 Donders Institute for Brain Cognition and Behavior, Department of Neurology, Center of Expertise for Parkinson & Movement Disorders, Radboud University Medical Centre, Nijmegen, The Netherlands; 2 Department of Neurology, Leiden University Medical Centre, Leiden, The Netherlands; 3 Department of Internal medicine, Division of Vascular medicine, Radboud University Medical Centre, Nijmegen, The Netherlands; 4 Department of Geriatric medicine, Radboud University Medical Centre, Nijmegen, The Netherlands; 5 Stichting Epilepsie Instellingen Nederland (SEIN), Hoofddorp, The Netherlands; 6 UCL Queen Square Institute of Neurology, University College London, London, UK

**Keywords:** Supine hypertension, Orthostatic hypotension, Syncope, Blood pressure, Feasibility, Implementation, Autonomic failure, Nocturia

## Abstract

**Background:**

In persons with Parkinson’s Disease (PD) or certain forms of atypical parkinsonism, orthostatic hypotension is common and disabling, yet often underrecognized and undertreated. About half of affected individuals also exhibit supine hypertension. This common co-occurrence of both orthostatic hypotension and supine hypertension complicates pharmacological treatments as the treatment of the one can aggravate the other. Whole-body head-up tilt sleeping (HUTS) is the only known intervention that may improve both. Evidence on its effectiveness and tolerability is, however, lacking, and little is known about the implementability.

**Methods:**

In this double-blind multicenter randomized controlled trial (phase II) we will test the efficacy and tolerability of HUTS at different angles in 50 people with PD or parkinsonism who have both symptomatic orthostatic hypotension and supine hypertension. All participants start with one week of horizontal sleeping and subsequently sleep at three different angles, each maintained for two weeks. The exact intervention will vary between the randomly allocated groups. Specifically, the intervention group will consecutively sleep at 6°, 12° and 18°, while the delayed treatment group starts with a placebo angle (1°), followed by 6° and 12°. We will evaluate tolerability using questionnaires and compliance to the study protocol. The primary endpoint is the change in average overnight blood pressure measured by a 24-hour ambulatory blood pressure recording. Secondary outcomes include orthostatic blood pressure, orthostatic tolerance, supine blood pressure, nocturia and various other motor and non-motor tests and questionnaires.

**Discussion:**

We hypothesize that HUTS can simultaneously alleviate orthostatic hypotension and supine hypertension, and that higher angles of HUTS are more effective but less tolerable. The Heads-Up trial will help to clarify the effectiveness, tolerability, and feasibility of this intervention at home and can guide at-home implementation.

**Trial registration:**

ClinicalTrials.gov NCT05551377; Date of registration: September 22, 2022.

## Background

Autonomic dysfunction is common, debilitating and often underrecognized in Parkinson’s Disease (PD) [1, 2]. The risk of orthostatic hypotension increases with age and disease duration. Up to one third of all people with PD experience orthostatic hypotension at some point during their disease course [2]. Orthostatic hypotension is also common amongst certain types of parkinsonism, especially in multiple system atrophy (MSA) with prevalence of up to 80% [3, 4]. In both PD and MSA, orthostatic hypotension is mostly neurogenic, but it may also be caused or aggravated by hypovolemia, dopaminergic drugs or other blood pressure (BP) lowering medications [1]. Orthostatic hypotension can present with orthostatic intolerance (e.g., postural light headedness), but the symptoms may also be less recognizable such as fatigue, cognitive slowing or coat hanger pain while standing. It is important to recognize and treat orthostatic hypotension as it may lead to syncope and falls with resulting injuries [5, 6]. The symptoms may also lead to a reduction in physical activity, which in turn aggravates other movement disorder symptoms such as balance and mobility problems [7], thereby increasing the risk of falling even further. Previous placebo-controlled randomized controlled trials (RCT) have shown that effective treatment of orthostatic hypotension increases physical activity [8], and improves functional mobility in people with orthostatic hypotension and PD or parkinsonism [9].

Up to half of all people with PD or MSA and orthostatic hypotension also exhibit supine hypertension [10]. Supine hypertension can be severe and last for several hours during nocturnal sleep, putting people at a higher risk for early morning hypertensive emergencies such as stroke and myocardial infarction [10–13]. Over time, the combination of the very high recumbent and very low upright BP may contribute to end-organ damage at the cerebral, cardiac and renal level [14, 15]. Indeed, among people with PD the presence of white matter lesions was associated with both supine hypertension and orthostatic hypotension [16]. Supine hypertension is also known to foster pressure natriuresis overnight thus promoting orthostatic hypotension [1, 13]. This may partially explain why orthostatic hypotension is often worse in the morning [17]. The common co-occurrence of orthostatic hypotension and supine hypertension makes pharmacological treatment very complex, as treatment of one aggravates the other [13].

A non-pharmacological and non-invasive intervention that can improve both orthostatic hypotension and possibly also supine hypertension is head up tilt sleeping (HUTS). The concept of HUTS is based on clinical observations made over 80 years ago [18–20]. These observations showed symptomatic and objective improvement of orthostatic hypotension during daytime. However, thus far HUTS has only been investigated in small and largely observational cohort studies, and never in a population with movement disorders [21–24]. The optimal tilt angle of HUTS is currently unknown but based on the presumed gravitational effect a steeper head-up tilt sleeping position is likely to be most effective, but is also less tolerable due to more discomfort in the sleeping position. The studied angles showing improved orthostatic tolerance varied from 12° to 40° [18, 19, 21–23], yet these studies did not evaluate the impact on nocturnal supine hypertension. This could be attractive, however, because from a physiological perspective, one would expect a more marked effect on supine hypertension rather than on orthostatic hypotension. HUTS will likely alleviate supine BP due to direct gravitational effects while orthostatic BP improvement is mediated by changes in extracellular fluid compartments. Accordingly, a placebo controlled RCT applying low HUTS angles (5°) found no effect on orthostatic hypotension, but more frequent occurrence of ankle oedema in the intervention group. This suggests that even a modest angle has potential to reduce supine hypertension [25, 26]. In clinical practice, HUTS is often not recommended, and when it is modest tilt angles are suggested with presumably at best also modest effects, as a guideline on practical implementation is still lacking [25, 26].

We here describe the design of the Heads-Up study, in which we investigate the potential efficacy and tolerability of different angles of HUTS as a treatment for both supine hypertension and orthostatic hypotension in people with PD. We will evaluate the effect of different angles of HUTS on several BP outcomes, orthostatic intolerance, compliance, tolerability, nocturia, as well as motor- and non-motor PD symptoms. Finally, we will explore whether certain participant characteristics may predict the effectiveness of HUTS.

## Methods

### Study design

**Fig. 1:**
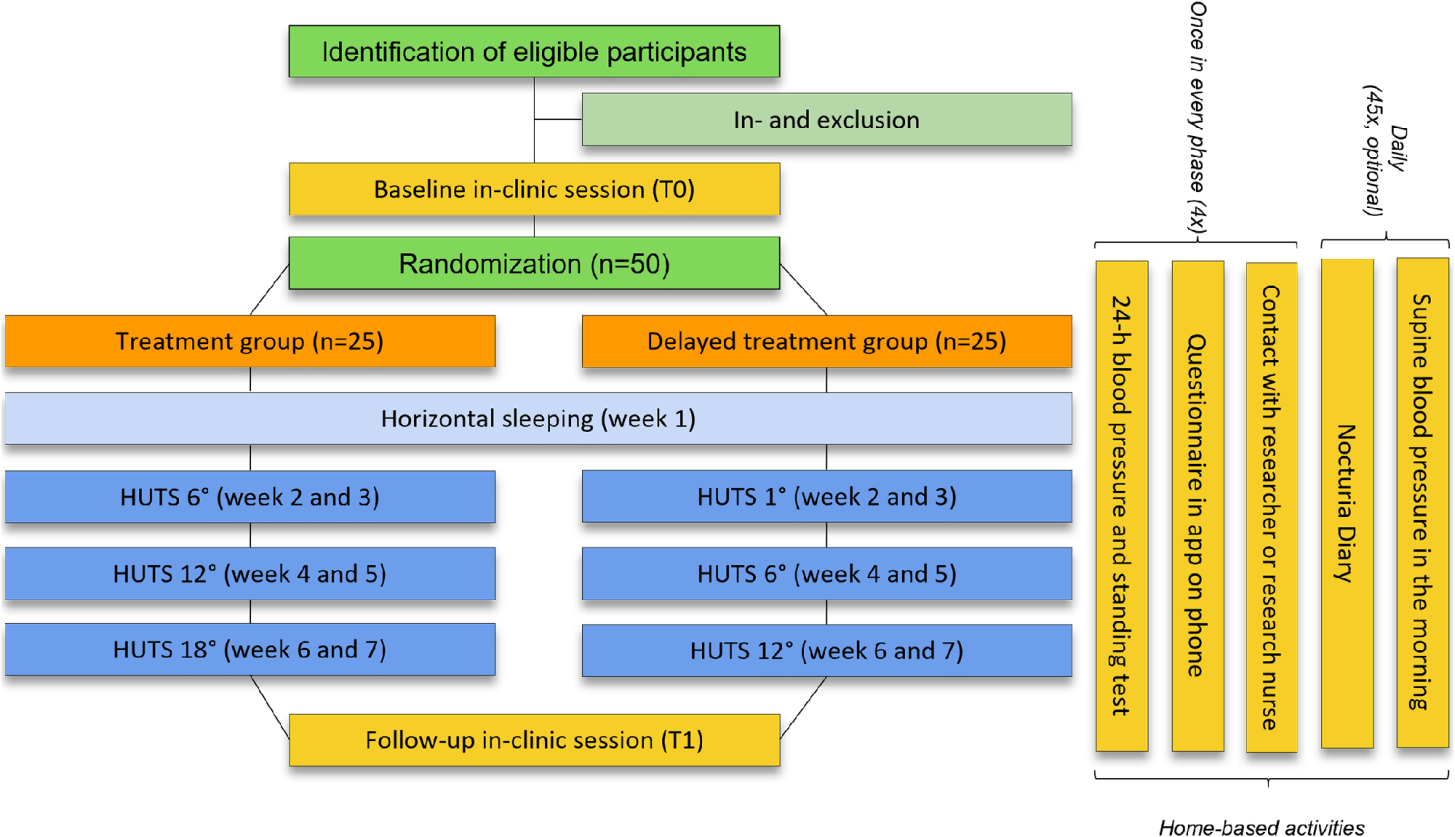
Overview of the trial. HUTS, head-up tilt sleeping

The Heads-Up trial is a double-blind, phase II RCT. Participants will be randomized in two groups: the treatment group and the delayed treatment group (Fig. 1). It is a two-center study performed at the Radboud University Medical Center (Radboudumc) and Leiden University Medical Center (LUMC), both located in The Netherlands. The total study duration for participants is seven weeks.

#### Population

We aim to include a diverse population of fifty adults diagnosed with PD or parkinsonism by a neurologist that have both supine hypertension (systolic BP of ≥ 140 mmHg, and/or diastolic BP of ≥ 90 mmHg, after 5 min of supine rest) [12] and orthostatic hypotension (systolic decrease of ≥ 30 mmHg [27] or diastolic decrease of ≥ 10 mmHg upon standing, i.e. the orthostatic hypotension criteria for those with co-existing supine hypertension) [27, 28]. Participants must experience symptoms of orthostatic intolerance (e.g., dizziness, cognitive slowing or blurry vision while standing). Participants must be able to walk, with or without walking aid, must have a stable medication regime for both supine hypertension and orthostatic hypotension during participation, and are not allowed to simultaneously participate in other intervention trials. Finally, participants are only eligible if they can adhere to the study schedule themselves or with help of support at home.

#### Recruitment

We will recruit primarily at our outpatient clinics (Radboudumc & LUMC). In addition, other neurologists, Parkinson nurses, geriatricians and internists throughout the Netherlands will be invited to refer potentially eligible patients. We will also use open recruitment via social media and the media channels of the Dutch Parkinson Patient Association and ParkinsonNext (https://www.parkinsonnext.nl/), a platform connecting people with PD and an interest in research with researchers (n > 2000).

#### Procedure

Those who expressed their interest in participating are contacted by the research team to screen for eligibility and informed about the study protocol. If BP data is unavailable, we will discuss how these measurements can be obtained (self-measurements or with help from a researcher). All participants will receive elaborative explanation on the study procedures at the start of the first in-clinic session, and prior to participation they will sign an informed consent form. During the study duration of seven weeks there will be at least seven scheduled moments of contact. Participants will perform several activities and measurements:

- First, we will plan an in-clinic visit (either at the Radboudumc or LUMC). For consistency, this visit will always be scheduled in the early afternoon. Here, participants can ask additional questions and sign informed consent. The researchers will then gather baseline characteristics, perform their initial assessments with continuous BP measurements and several questionnaires. The assessments and questionnaires can be found in Table 1. Finally, the participant will be asked to install an app through which they can report their at-home measurements.
- After this session, the participants are randomized to one of the two groups. We will apply the randomization feature of the data management system Castor EDC with block sizes of two and four to allow the interim analysis. We will stratify based on gender. The researchers who perform the assessments and/or are involved in the analyses will remain blinded to the participant allocation during the study. Only the researchers who perform the randomization and deliver the materials at home will know which group the participants are allocated to. We will not inform the participants that we expect that the first angle in the delayed treatment group has no effect on BP regulation, making this a double-blind study.
- We will schedule an appointment to deliver all study materials within one week after the first session.
- Participants will perform daily at home measurements of supine BP and their weight. They will be asked to record their measurements in the app.
- We will schedule four video calls with the participants to discuss potential caveats and supervise the BP standing tests. Participants will also be asked to fill out several questionnaires in the app on this day and to disclose whether they slept in the prescribed angle. During this video check-in we will also guide the initiation of the 24-hour ABPM (Table 1).
- Participation ends with a follow-up in clinic session, also scheduled in the early afternoon for consistency, where the BP measurements and questionnaires from the first in clinic session are repeated and participants are asked about their experiences. When they want to proceed HUTS, they are offered to keep the materials and to do so under supervision of their primary care physician.

#### Intervention

All participants in this study are subject to a six-week intervention. They will be sleeping in a whole-body HUTS position at three different angles, for two weeks each. The treatment group will sleep at HUTS angles of 6°, 12° and 18°. The delayed treatment group will first sleep at the placebo angle of 1° which is considered the control, after which they also sleep at 6° and 12° HUTS (Fig. 1). In both groups the intervention is preceded by a week of horizontal sleeping for baseline measurements.

**Fig. 2:**
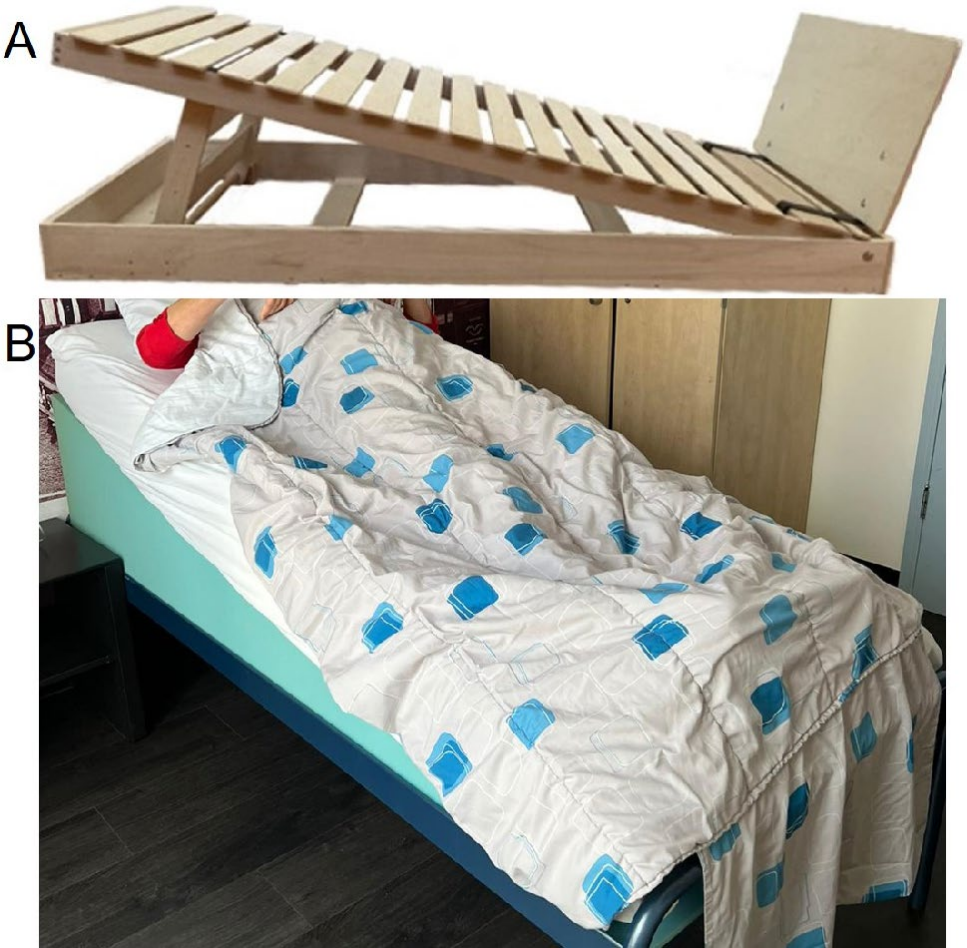
Image of the HUTS method for in the participant’s home. In both situations a 12° angle is shown. **A**) The wedge-shaped mattress can be placed underneath the participant’s mattress or used separately. **B**) The frame can easily be adapted to fit to all angles that are prescribed during the study

The necessary materials will be delivered to the participants’ homes. To facilitate implementation in the home situation we have developed a frame that can be used to tilt the mattress into all different angles (Fig. 2A). For those who do not wish to use this frame, we offer wedge shaped mattresses with similar HUTS angles (Fig. 2B). The effect of HUTS on sleep quality is not yet known, but higher angles of HUTS may cause discomfort. Slipping can be reduced by increasing the friction of bedcover fabric, placing a rolled-up towel under the hips or in several other ways. We will expand this list as participants figure out what works for them during the study. We offer to provide each participant with a sleeping partner the frame for both so that they can sleep next to each other in the same angle during the trial. For good application of the HUTS method in the participant’s home, we will pay a home visit not only to deliver the materials, but also to offer support. For those who require or request extra help during the study we will provide this by offering a telephone or video call and, if necessary, by additional home visits.

#### Testing of materials

We organized several test sessions for patient researchers to test different HUTS methods and to provide feedback on the design and usability. From these preparatory sessions we learned that it is not feasible to apply the intuitive method of blocks underneath the headboard of the participant’s bed as this is very unstable at the higher angles. The final frame (Fig. 2A) comes with a footboard to prevent participants from sliding down at the higher angles. This reduces the risk of falls from the bed during the night. We also supply handrails on the side of the bed for easier turning, as difficulties with turning is a common problem in people with PD. These handrails, together with the footboard, also form a safety barrier that prevents participants from sliding or falling out of bed and can help them get out of bed safely. For the safety of the participants, we decided to implement the highest two angles (12° and 18°) by placing the frame on the floor. We did realize that this may be problematic or even hinder participation for those who do not have the space at home for this additional frame on the floor, but we considered the alternative unsafe.

### Outcome measures

#### Primary outcomes

##### Efficacy

The primary outcome is the home-based overnight supine BP recorded four times with the 24-hour ambulatory blood pressure measurement (ABPM). This recording will be done during one of the last three days of every phase. The measurements during the baseline horizontal week will be used to calculate the change score to determine the effect of each angle on the BP. The BP device will measure the BP every half hour during the night, according to the guidelines for ABPM measurements [29]. Participants will self-report the actual time they spend lying down in bed.

**Table 1:** Overview of study procedures

Activity	In-clinic 1	Wk1	Wk2-3	Wk4-5	Wk6-7	In-clinic 2
Informed consent	X					
Demographics	X					
Tilt-table and BP standing test	X					X
MDS-UPDRS	X					X
Questionnaires OHQ, PSQI, FES, selected questions from SCOPA, PDQ-39, HADS and MHC-SF	X					X
Timed Up and Go test	X					X
Average overnight and daytime BP with 24-h ABPM (4x)		X	X	X	X	
Supine BP (daily, 45x)		X	X	X	X	
Standing BP (guided, 4x)		X	X	X	X	
Questionnaire on phone app (4x) - OHQ - ICIQ-N - Number of falls - Nocturia - Subjective comfort of HUTS		X	X	X	X	
Nocturia measurements - Body weight (45x) - Nighttime urine production (4x)		X	X	X	X	
Interview on barriers and facilitators of HUTS						X
**Protocol**						
Instructions	X					
Sleeping horizontally		X				
(Possible) Home visit to install angle		X	X	X	X	
HUTS angle 1			X			
HUTS angle 2				X		
HUTS angle 3					X	
Phone/video call with researcher		X	X	X	X	
Aftercare (personalized advice)						X

*BP = blood pressure; HUTS = sleeping in head-up tilt; MDS-UPDRS = Movement Disorders Society Unified Parkinson Disease Rating Scale; OHQ = orthostatic hypotension questionnaire; PSQI: Pittsburgh sleep quality index; FES: Falls Efficacy Scale; SCOPA: SCales for Outcomes in PArkinson’s disease; PDQ: Parkinson’s disease questionnaire; HADS: Hospital anxiety and depression scale; MHC-SF: Mental Health Continuum Short Form; ICIQ-N: International Consultation on Incontinence Questionnaire Nocturia Module*

##### Tolerability

We will evaluate the tolerability of HUTS with four indicators: (1) compliance, as measured by the daily question if they slept in the right angle, presented as proportion of participants that were > 80% of the study period compliant to the prescribed intervention; (2) the proportion of participants who did not tolerate the angle and returned to the previous angle; (3) the number of dropouts and if provided their reason for dropping out of the study, and; (4) reported barriers and motivators for using HUTS (evaluation during the final in-clinic session).

##### Secondary outcomes

The first secondary outcome is the daily supine morning BP. This is measured and reported by the participants themselves before taking a seated position in bed.

During the four video sessions data is gathered on orthostatic BP through supervised home-based standing tests and daytime BP measured by the 24-h ABPM, which records daytime BP three times per hour. Other ABPM parameters will be considered as well (e.g., BP variability and nocturnal dipping).

Besides BP, the symptoms of orthostatic intolerance are determined with the Orthostatic Hypotension Questionnaire (OHQ) [30] and the cardiovascular questions of the Scales for Outcomes in Parkinson’s disease – Autonomic symptoms (SCOPA-AUT) [31]. Nocturia will be quantified during the ABPM measurement by collecting and reporting the total volume of urine produced during the night. On all other days, the overnight weight loss will be used to estimate the total volume lost. The International Consultation on Incontinence Questionnaire Nocturia Module (ICIQ-N) is used to determine the amount of bother experienced as a result of nocturia [32].

At both in-clinic sessions participants will be subjected to a phased tilt table test protocol including heart rate and beat-to-beat BP recordings (Finapres Medical Systems, Enschede, The Netherlands). We will tilt from a horizontal position to 15°, 30°, 45° and finally 60°. This will provide us with systematic measures of BP responses to different degrees of orthostatic positions. This, together with the standing test, will be used to investigate whether clinically relevant predictive values for the effectiveness of HUTS can be identified. Adverse events will be registered and grouped per treatment phase and group.

We selected several questionnaires which will be used to monitor the wellbeing of the participants in multiple areas:

- The Pittsburgh Sleep Quality Index (PSQI) [33] to investigate sleep quality, duration and parasomnias, the number of falls during participation, and the fear of falling as determined by the Falls Efficacy Scale (FES) [34].
- The Parkinson’s Disease Questionnaire (PDQ-39) to evaluate mobility, activities, emotional wellbeing, stigma, social implications, cognitive impairment and bodily discomfort [35].
- The Hospital Anxiety and Depression Scale (HADS) [36] and the Mental Health Continuum-Short Form (MHC-SF) [37] to monitor the emotional wellbeing of the participants.

### Analysis

#### Interim analysis

The interim analysis will be used to provide information for a sample size calculation for a (phase III) follow-up study. No preliminary results with relation to the outcome of the study will be calculated. The average overnight BP from the first twelve participants will be used in this analysis. For these participants the change score comparing baseline (0°, week 1) and the 12° angle (week 5 or 7) will be determined, and only the average, standard deviation and confidence interval will be calculated. The researchers that are in contact with the participants will remain blinded to treatment groups, therefore this analysis will be performed by a non-blinded member of the research team. We will not use this analysis to terminate the study.

#### Final analysis

After completion of the study the data will be analyzed according to the intention-to-treat principles. For the main analysis of the overnight BP measured with the 24-hour ABPM in each phase we will use the overnight BP measured in week 1 as baseline to determine the change score. The 1° angle will serve as placebo, and the rest of the HUTS angles are grouped together for overall effectiveness and the increasing angles separately for determining which angle is the most effective for reducing the overnight BP. To estimate the effect of the HUTS angle we will use a linear mixed model with as a dependent variable the change in overnight BP as measured during the 24-hour ABPM, with fixed effects for angle, group and visit and with a random effect for subject. As covariables the baseline BP value, age and disease duration will be used. The within group differences will also be analyzed for all angles with a linear mixed effects model per group. Dependent variables in this calculation are the angle (fixed effect) and subject (random effect). The daytime variation will be calculated in the same way as described for the overnight BP.

The secondary outcome morning supine BP will be analyzed by averaging the three consecutive morning measurements and comparing the 13 timepoints from each phase with the baseline for overall effect, and by comparing each of the three phases to investigate the difference in effect for angles taking into account the time-effect.

Additional explorative analyses based on the per-protocol principles will be performed, this includes the analysis on tolerability of HUTS, baseline characteristics collected at the in-clinic sessions, the results from the PSQI, falls, FES, PDQ-30, HADS and MHC-SF which all will be exploratively analyzed. The tilt table test will be analyzed to investigate the differences between responders and non-responders by looking at the severity of supine hypertension and orthostatic hypotension.

### Sample size calculation

Since this is a phase II clinical trial, no formal sample size was calculated. We aim to study the effect of HUTS on clinical outcomes to power a future phase III RCT. We expect a large effect of the intervention on the main study parameters, meaning that 50 participants will be sufficient for this RCT [38].

### Data monitoring

This study will be monitored by an independent monitor through several on-site visits. No serious adverse events are expected, therefore no study termination points are identified beforehand. We will not install a data safety monitoring board, and no auditing will occur. The trial will be coordinated and managed from the Radboudumc.

## Discussion

We present the rationale and design of the Heads-Up trial, a double-blind phase II RCT to determine the potential benefit of different angles of HUTS as a treatment for both supine hypertension and orthostatic hypotension in people with PD and parkinsonism. Although the HUTS concept has been known for almost 80 years, many unknowns persist regarding the efficacy and feasibility. We therefore propose a home-based trial with a strong focus on the implementability.

Although HUTS is perceived as a simple method, there are several practical challenges which we tried to tackle in advance as much as possible. These can be found in the Methods section. The present study also focuses in part on investigating these challenges and hence the tolerability of the HUTS method. It may be difficult to acclimate to sleeping in a tilted position and all the adjustments that need to be made to implement it. The practical application is complex and highly individual, requiring a personalized approach. Measures will be taken to ensure that space or sleeping situations do not lead to an inclusion bias. To ensure this, we will provide each individual participant with all necessary support, including at least one home-visit for installation.

Apart from the practical challenges of the study, there are several important methodological issues. With the specific population studied here it may prove difficult to include a diverse group of participants (e.g., gender or with relation to socio-economic status), as men are more likely to be diagnosed with PD or parkinsonism, and underserved populations (such as those with a migration background) are often not reached. Among persons with PD, autonomic failure usually develops late in the course of the disease. This might impede recruitment due to frailty and abundant physical and cognitive symptoms. However, persons with parkinsonism, specifically those with MSA, often exhibit orthostatic hypotension at an earlier disease stage, sometimes even as the main presenting symptom. Although their disease progression may be faster, we expect that we will be able to recruit more mobile participants among these subgroups. To include underserved populations, we will recruit not only through neurologists in the outpatient clinics of university medical centers, but also in smaller or rural hospitals and clinics. We will also reach out to specialized nurses, physiotherapists, and people with PD themselves through open recruitment.

An additional methodological challenge due to the design of the study is the complexity of the statistical analyses. The order of the angles of the intervention are not randomized, which was chosen due to the impact that the order may have on the perception of the different angles. By increasing the tilt angles step by step, the participants can slowly get used to tilted sleeping. We hope this improves the tolerability of the higher inclinations and reduces dropouts due to uncomfortable sleeping. If they still do not tolerate a new, higher angle, we will ask participants to return to the previous angle. From a physiological perspective one would expect that the impact HUTS will increase proportionally to the size of the angle. Randomizing the order of the angles would require wash-out periods to evaluate the independent effect of each angle but also longer intervention periods for steeper angles to reach a steady state of the effect. We therefore preferred the fixed and incremental order of HUTS inclination as a more practical design. The intention-to-treat analysis is likely to influence our results for the efficacy of the higher angles; we will therefore perform an additional per-protocol analysis.

Orthostatic hypotension often results from multiple contributing factors that can be neurogenic and non-neurogenic. Autonomic dysfunction, nocturnal hypertension, nocturia, hypovolemia, BP medication or dopaminergic medication may all contribute to orthostatic hypotension, but we cannot study each factor separately or their interaction with each other. We will try, however, to identify hemodynamic markers to predict HUTS efficacy. We will also monitor the impact of HUTS on nocturia, an often neglected and incapacitating symptom in PD or parkinsonism. Interestingly, nocturia seems more prominent in persons with supine hypertension and may contribute to orthostatic intolerance, but no concise evidence exists [17]. By monitoring nocturia and including those with supine hypertension and orthostatic hypotension, we hope to uncover the complex interplay between these factors.

Taken together, HUTS is an attractive intervention with the unique potential to positively impact supine hypertension and orthostatic hypotension simultaneously. Although the intervention seems simple and straightforward, the best way to implement in often frail people with movement disorders needs further study. If the current trial proves successful, a definitive phase III RCT will be designed, powered to study clinically relevant outcomes. The current study will help to determine which angles and target population this new trial should focus on. The current work will lay the foundation for practical guidelines for a structured and personalized application of HUTS.

### Trial status

On the 18th of February 2023 the first participant was included in this study. Currently (November 2023) 12 participants have finished their participation, and there are 3 active enrolled participants. The trial is currently recruiting. The last visit is expected to be completed in June 2024.

## Data Availability

The final anonymized dataset and analyses will be made available through the Radboud Data Repository and will be shared with the sponsor.

## Abbreviations

ABPM: Ambulatory blood pressure measurement
BP: Blood pressure
HUTS: Head up tilted sleeping
LUMC: Leiden University Medical Centre
MDS-UPDRS: Movement Disorder Society-Unified Parkinson’s Disease Rating Scale
MSA: Multiple system atrophy
PD: Parkinson’s disease
Radboudumc: Radboud University Medical Centre
RCT: Randomized controlled trial
TUG: Timed Up and Go
